# Characterisation of the tryptophan synthase alpha subunit in maize

**DOI:** 10.1186/1471-2229-8-44

**Published:** 2008-04-22

**Authors:** Verena Kriechbaumer, Linda Weigang, Andreas Fießelmann, Thomas Letzel, Monika Frey, Alfons Gierl, Erich Glawischnig

**Affiliations:** 1 Lehrstuhl für Genetik, Technische Universität München, D-85350 Freising, Germany; 2 Analytische Forschungsgruppe des Lehrstuhls für Chemie der Biopolymere, Technische Universität München, D-85350 Freising, Germany

## Abstract

**Background:**

In bacteria, such as *Salmonella typhimurium*, tryptophan is synthesized from indole-3-glycerole phosphate (IGP) by a tryptophan synthase αββα heterotetramer. Plants have evolved multiple α (TSA) and β (TSB) homologs, which have probably diverged in biological function and their ability of subunit interaction. There is some evidence for a tryptophan synthase (TS) complex in Arabidopsis. On the other hand maize (*Zea mays*) expresses the TSA-homologs BX1 and IGL that efficiently cleave IGP, independent of interaction with TSB.

**Results:**

In order to clarify, how tryptophan is synthesized in maize, two TSA homologs, hitherto uncharacterized *Zm*TSA and *Zm*TSAlike, were functionally analyzed. *Zm*TSA is localized in plastids, the major site of tryptophan biosynthesis in plants. It catalyzes the tryptophan synthase α-reaction (cleavage of IGP), and forms a tryptophan synthase complex with *Zm*TSB1 *in vitro*. The catalytic efficiency of the α-reaction is strongly enhanced upon complex formation. A 160 kD tryptophan synthase complex was partially purified from maize leaves and *Zm*TSA was identified as native α-subunit of this complex by mass spectrometry. *Zm*TSAlike, for which no *in vitro *activity was detected, is localized in the cytosol. *Zm*TSAlike, BX1, and IGL were not detectable in the native tryptophan synthase complex in leaves.

**Conclusion:**

It was demonstrated *in vivo *and *in vitro *that maize forms a tryptophan synthase complex and *Zm*TSA functions as α-subunit in this complex.

## Background

Tryptophan is an essential amino acid for human nutrition. In kernels of cereals, e.g. maize (*Zea mays*), the tryptophan content is low, limiting the nutritional value. Significant effort is made to breed maize lines with enhanced tryptophan content [1,2]. In addition to its function as protein component, plants utilize tryptophan as precursor of a large variety of secondary metabolites like terpenoid indole alkaloids, indole glucosinolates, and indolic phytoalexins (reviewed in: [3,4]). Of special importance is the tryptophan-derived plant hormone indole-3-acetic acid (IAA), which is involved in numerous processes, including embryo development, apical dominance, and tropisms [4,5]. These essential functions of tryptophan emphasize the need to understand its synthesis in plants in more detail.

**Figure 1:**
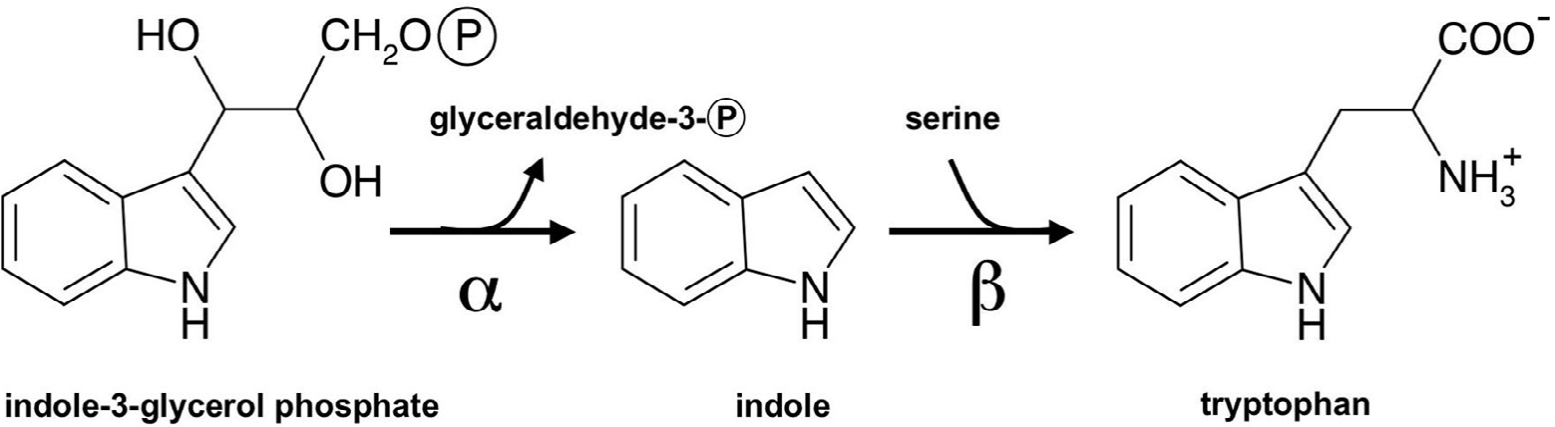
**General scheme of the tryptophan synthase reaction.** Indole, which is formed from IGP by the α-subunits is channelled to β-subunits, which synthesize tryptophan from indole and serine.

In bacteria, such as *Escherichia coli *and *Salmonella typhimurium*, tryptophan is synthesized from indole-3-glycerol phosphate (IGP) by a tryptophan synthase (TS) complex [6]. IGP is cleaved by the TS α-subunits (TSA) to indole and glyceraldehyde-3-phosphate (α-reaction). Then indole is transported via a 30 Å intermolecular tunnel to the tryptophan synthase β-subunits (TSB) that catalyze the condensation of indole and serine (β-reaction) to tryptophan (Figure 1; for review, see [7,8]). This substrate channelling ensures that indole does not escape from the enzyme complex. The reaction mechanism of this bacterial αββα complex has been studied in great detail. The α- and β-subunits interact in a highly cooperative manner and regulate each other reciprocally by allosteric interactions. In addition, alternative TSBs that are highly active, independent of interaction with the unique TSA, are expressed in some prokaryotes [9]. In fungi interaction of TSA and TSB is obligate as both functions are present on a single polypeptide [9,10].

The picture is more complex in higher plants. The homologs of the bacterial *TSA *and *TSB *genes are generally duplicated, e.g. the *Arabidopsis thaliana *genome contains two putative *TSA *and four putative *TSB *genes. Currently the role of these different isoforms is not fully understood. Functional relevance of *At*TSA1 (At3g54640) and *At*TSB1 (At5g54810) was demonstrated by the facultative tryptophan auxotroph mutants *trp3 *and *trp2*, respectively [11,12]. *At*TSA1 and *At*TSB1 are both localized in the plastid [11,13,14]. Based on immunoaffinity chromatography it was strongly suggested that the two proteins form an active αβ complex [14]. However, it is not known whether formation of such complexes is a general phenomenon in plants.

Among the cereals most information on TSA and TSB homologs is available for maize: Two highly similar *TSB *genes (*ZmTSB1 *and *ZmTSB2*) have been identified, sharing 96% identity on the mature protein level. *Zm*TSB1 and *Zm*TSB2 are functionally redundant active TSB enzymes. While single mutations in either gene do not affect tryptophan synthesis, the double mutant *orange pericarp *(*orp1 *= *Zmtsb1*, *orp2 *= *Zmtsb2*) is tryptophan auxotroph [15,16]. Four TSA homologs are present: BX1, IGL, *Zm*TSAlike [17], and *Zm*TSA (this work). BX1 is essential for providing indole as precursor of the natural pesticide 2,4-dihydroxy-7-methoxy-2H-1,4-benzoxazin-3(4H)-one (DIMBOA) [18]. The 3-dimensional BX1 structure has been determined and shown to be very similar to the active conformation of bacterial TSAs [19]. *Igl *transcription is triggered by insect feeding and IGL provides indole as a volatile signal for parasitic wasps [17,20]. BX1 and IGL efficiently cleave IGP to form indole, while the activity of bacterial TSA subunits is dependent on the interaction with a β-subunit [17,18]. These enzymatic properties, their specific transcriptional regulation, and the lack of growth defects of *bx1 *and *igl *mutant plants suggested that additional TSA-homologs are involved in tryptophan biosynthesis.

In this study we aimed to identify the TSA homolog from maize that is involved in tryptophan biosynthesis. *Zm*TSA is catalytically active and interaction of *Zm*TSA with a β-subunit strongly enhances the catalytic efficiency of the α-reaction. A protein purification strategy was applied to obtain direct evidence that angiosperms, similarly to bacteria, form a tryptophan synthase complex.

## Methods

### Plant material and growth conditions

The following maize (*Zea mays*) lines were analyzed: B73 (wildtype), *bx1 *mutant [21], and the *tsb *mutants (*orp1 +*/*orp1 orp2*) and (*orp1 orp2*/*+ orp2*) [15] that were kindly provided by the maize genetic stock center. Seedlings were germinated in a beaker rolled in wet filter paper (603/N, 75 g m^-2^, Sartorius) at 28°C in the dark and after three days the seedlings were transferred to soil and incubated in a Heraeus HPS 2000 growth chamber (light: 100 μmol m^-2 ^s^-1^; 16 h/d).

### Identification of ZmTSA and heterologous expression of standard proteins

For isolation of cDNAs a library prepared from 12 day old seedlings, line *bx1 *[21] was used [17]. A *ZmTSAlike *cDNA clone was isolated based on the genomic sequence [17] and confirmed to be full-length by RACE. The *ZmTSA *cDNA was cloned [GenBank:EU334442] based on the EST AY107255 (gene bank) and the EST-TUG *Zm*tuc03-08-11.4557 (maize genomic database) and confirmed to be full-length based on the genomic sequence. *Zm*TSA and *Zm*TSAlike were analyzed for plastid targeting sequences using the programs "TargetP" and "iPSORT" [22,23].

BX1 and IGL expression and purification have been described previously [17,18]. For heterologous expression of *Zm*TSA and *Zm*TSAlike, an NdeI/BglII-fragment, for *Zm*TSB1 expression an NdeI/BamHI-fragment of the coding sequence excluding plastid-targeting sequences was amplified by PCR. The following primers were used: *Zm*TSA: GCATATGCCGCGCAGCATCTCCG, TCTTACGCTCTTTGCTAACGAAAATGG; *Zm*TSAlike: CGCATATGGCCAACGGCGGCG, GGGAGTGAGATCTGCTCACGGC; *Zm*TSB1: CATATGGCGGCCTCCCCCGCTGCCG, CTCGGATCCAGCCCTCCTCTCCGGTG. The coding sequences were cloned into pET28a His-tag vector, heterologously expressed, and purified under native conditions by His-tag affinity purification via Ni-NTA agarose according to the manufacturers' suggestions (Qiagen, Hilden, Germany).

For the detection of TSA/TSB complex formation *in vitro*, size exclusion chromatography (HiLoad™16/60 Superdex™200 prep grade, Amersham Biosciences, Little Chalfont, UK) was performed using 100 mM Tris-HCl, pH 8.0, 100 mM KCl at 0.5 ml min^-1 ^and 20°C. The column was calibrated using the protein standards cytochrome c (12.4 kD), carbonic anhydrase (29 kD), bovine albumin (66 kD), alcohol dehydrogenase (150 kD), and β-amylase (200 kD).

### Transcription analysis

For detection of *ZmTSA *and *ZmTSAlike *expression total RNA was isolated from the wildtype line B73 and quantitative real time PCR was carried out using the LightCycler/Syb^®^-Green dye system (Roche, Mannheim, Germany) with the following primer pairs: *ZmTSA*: CACTGCTGGAGACCCTGACT, GGTTCATGGCAATGCGGCCT; *ZmTSAlike*: CCACAAAGGCAGCGCTCGGAGGTG, GCCTCGCTCCTCAGCAACGTCGTCT; *GAPDH C*: GCTAGCTGCACCACAAACTGCCT, TAGCCCCACTCGTTGTCGTACCA.

Tissues analyzed are the following: leaf from 12 d old plants (12 d leaf), 12 d leaf methyl jasmonate treated, 12 d leaf elicitor treated [20], 4 d shoot dark grown, 6 d shoot dark grown, 6 d shoot light grown, 3 week root, 10 week crown root, 8 week stem, 10 week leaf, husk, silk, cob, tassel, kernel 1 week after pollination (wap), kernel 3 wap.

### Tryptophan synthase activity assays

Plant protein fractions (200 μg) or purified recombinant enzyme (2 μg) were incubated 3 h for plant protein, 5 min for recombinant proteins, respectively, at 30°C, in 80 mM potassium phosphate buffer, pH 8.2 containing the following substrates: α-reaction: 100 μM IGP [24]; β-reaction: 50 μM indole, 60 mM L-serine, 50 μM pyridoxal phosphate; αβ-reaction: 100 μM IGP, 60 mM L-serine, 50 μM pyridoxal phosphate, concentration ranges were analyzed for determination of kinetic parameters.

The products indole and tryptophan were quantified by HPLC (RP-column: LiChroCART 125–4, RP-18, 5 μm; Merck, West Point, PA) using diode array (PDA-100, Dionex, Idstein, Germany) and fluorescence detection (RF-10A_XL_, Shimadzu, Duisburg, Germany; excitation: 285 nm, emission: 360 nm). The mobile phase was delivered with a flow rate of 1 ml min^-1 ^with an initial mixture of 15% (v/v) MeOH in 0.3% (v/v) HCOOH followed by a 15 min linear gradient to 100% MeOH.

### Plant protein purification

Leaf tissue (50 g) was homogenized in liquid nitrogen and extracted in 5 ml 50 mM Tris-HCl, pH 8.0, containing 10 mM EDTA, 5 mM DTE, 1 mM PMSF, and 10% Polyclar AT (Serva, Heidelberg, Germany) and centrifuged 20 min at 10.000 g (4°C). The supernatant was subjected to an anion exchange column (MonoQ HR 5/5, Amersham) equilibrated with 100 mM Tris-HCl, pH 8.0, 10 mM EDTA, 5 mM DTE, 100 mM NaCl at 4°C. The column was then washed with 10 Vol of the same buffer and eluted with 100 mM Tris-HCl, pH 8.0 containing 10 mM EDTA, 5 mM DTE, and 1 M NaCl in a 20 Vol linear gradient. Fractions around 450 mM NaCl showed TS activity and were subjected (0.5 ml min^-1^, 20°C) to gel permeation chromatography (HiLoad™16/60 Superdex™200 prep grade, Amersham, equilibrated with 100 mM Tris-HCl, pH 8.0 including 100 mM KCl). The column was eluted with the same buffer (180 ml, 0.5 ml min^-1^). 1 ml fractions were collected and tested for α- and β-activity. The 54 to 56 ml fractions were precipitated by addition of 10% TCA, redissolved in 10 μl 10 mM Tris-HCl, pH 6.8, 20 mM DTT, 2% (w/v) SDS, 0.01% (w/v) bromphenol blue, 10% (w/v) glycerol, and subjected to SDS-PAGE.

After fixation for > 2 h with a 40% (v/v) MeOH/10% (v/v) HOAc solution and washing in water for 2 × 10 min the gels were stained over night with Coomassie dye (0.08% (w/v) Coomassie Brilliant Blue G250, 1.6% (w/v) *ortho*-phosphoric acid, 8% (w/v) (NH_4_)_2_SO_4_, 20% (v/v) MeOH) and destained in 1% (v/v) HOAc [25]. The protein bands between 25 and 60 kD were cut out and the gel piece was further destained in a thermo mixer with water (2 × 30 min 37°C), 200 mM NH_4_HCO_3_, pH 7.8 (2 × 30 min 37°C), and 50% acetonitrile (ACN) (2 × 5 min 37°C). The gel slice was shrunk in 100% ACN, the liquid supernatant was removed and the gel dried in a SpeedVac for 5 min.

### Identification of tryptophan synthase protein components: sample preparation

Tryptic digestion: 100 μl trypsin solution (200 ng μl^-1^, Promega) were directly pipetted on the gel piece, incubated for 10 min on ice to allow the trypsin to move into the gel and then covered with 500 μl 25 mM NH_4_HCO_3 _followed by 16 h incubation at 37°C. Digestion was stopped by adding 50 μl of 10% trifluoroacetic acid and the supernatant was transferred to a new tube. Peptides were extracted by consecutive basic and acid extraction. Basic extraction: 50 μl 40 mM NH_4_HCO_3 _were added to the gel, shaken for 30 min at 37°C, and the supernatant transferred to a new tube. The same incubation followed after addition of 50 μl ACN. Both steps were repeated and the supernatants pooled. Acid extraction: The gel piece was extracted twice in 50 μl 5% (v/v) HCOOH for 30 min. The gel piece was shrunk twice in 50 μl ACN. All the collected supernatants were pooled, dried in a SpeedVac and dissolved in 100 μl 20 mM ammonium acetate buffer, pH 7.4, 10% (v/v) ACN, 5 mM DTT.

A further step of digestion was performed in solution (10 μl trypsin solution, 200 ng μl^-1^, 8 h, 37°C) to apply a maximum amount of hydrolyzed peptides without miscleavage. Latter is important for a reproducible identification of qualifying peptides.

### Liquid chromatography – mass spectrometry (LC-MS)

An Agilent micro HPLC system (series 1100, Waldbronn, Germany) consisting of a quaternary capillary pump (G1376A), a degasser unit (G1379A), an auto sampler (G1377A), and a column in a thermostat set to 40°C (G1316A) was used in combination with a single time-of-flight mass spectrometer (LC/MSD TOF, Agilent Technologies, Santa Clara, USA). The chromatographic separation was performed with a Zorbax SB C18 column (150 × 0.5 mm i.d.; 5 μm, Agilent Technologies, Santa Clara, USA) by 8 μl sample injections. Prior to injection, the trypsinized protein samples were mixed with 200 μl 20 mM NH_4_Ac, 10% ACN (v/v), 5 mM DTT, pH 7.4, sonified for 15 min, filtrated via HV filter, and stored in an autosampler vial. The HPLC separation flow rate was 50 μl min^-1^. At the beginning of each chromatographic run, the composition of the mobile phase was kept at 95% 20 mM NH_4_Ac/5% ACN (v/v), following a gradient to 20% 20 mM NH_4_Ac/80% ACN (v/v) within 5 min and this final value was held for 15 min.

MS measurements were performed in positive ionization mode with the mass spectrometer equipped by an ESI source. The applied MS parameter were as follows: 350°C drying gas temperature, 420 Lh^-1 ^drying gas flow rate, 20 psig nebulizer gas pressure, 4000 V capillary voltage, 60 V skimmer voltage and 215 V fragmentor voltage. The mass-range was set to 150 – 3200 m/z and data acquisition was 0.88 cycles/sec. The drying gas nitrogen was supplied by a nitrogen generator (nitrogen purity ≥ 99.5%, Domnick Hunter, Willich, Germany). The ChemStation software (Rev. B.01.01, Agilent, Waldbronn, Germany) was used for system control and the Analyst QS software (LC-MS TOF Software, Ver. A.01.00 (B663), June, 2004) for the data acquisition.

### Expression of *Zm*TSA- and *Zm*TSAlike-GFP-fusion proteins

To construct vectors for expression of GFP-fusion protein [26], the stop codon of the *Zm*TSA coding sequence was replaced by a BglII restriction site, using the following primers: 5'-CGACTACACCAAATGAAAGAATGGAG-3' (forward), 5'-CTCGAGAGATCTGGCAATGCGGCCTTCAGG-3' (reverse). The full size *Zm*TSA cDNA fragment, in which the stop codon was eliminated, was then cut from the vector with EcoRI/BglII and cloned into the EcoRI/BamHI sites of the pEZS-NL vector (D. Ehrhardt, Carnegie Institution). The *Zm*TSA-eGFP chimera was cut with EcoRI, blunted, cut with XbaI, and cloned into the SmaI/XbaI sites of the PvuII-deletion of pPCV_E_35_E _plant transformation vector. The same strategy was used for the construction of the *Zm*TSAlike-eGFP chimeric cDNA, with the primer pair: CAAGCTGGCATACATGGAC/GGTACCAGATCTGGCATAGCAGCCTTCATA.

### Transfection of maize protoplasts and confocal microscopy

Maize LG22 seedlings were grown on an 8 h dark, 20°C/16 h light, 26°C regime for 6 to 8 days and were then transferred to the dark for 3 days. Protoplasts were isolated from the second true leaves essentially as described previously [27,28]. Digestion was performed in 1% (w/v) cellulase R10, 0,5% (w/v) Macerozyme R10 (both from Yakult Honsha), 0.6 M mannitol, 10 mM MES, pH 5.7, 1 mM CaCl_2 _for 2 h at 28°C on a rotating shaker (40 rpm). After filtration through a 65-μm nylon mesh, the protoplasts were collected by 3 min centrifugation at 200 g, followed by centrifugation at 100 g in floating solution (25% sucrose (w/v), 10 mM MES, 20 mM KCl). Floating protoplasts were washed in 0.6 M mannitol, 4 mM MES, 20 mM KCl and counted. Electroporation was performed with 2.5 × 10^5 ^protoplasts and 40 μg of each plasmid in 300 μl 4 mM MES-KOH, pH 5.7, 0.6 M mannitol, 20 mM KCl. Transformed protoplasts were incubated in the dark at 25°C for 20 h in 4 mM MES-KOH, pH 5.7, 0.6 M mannitol, 4 mM KCl.

Confocal microscope images were taken using an Olympus FV1000 confocal laser microscope with a 40× water objective. The excitation wavelength for eGFP detection was 488 nm.

## Results

### Isolation of *Zm*TSA

In maize, four genes encoding TSA homologs are present. *Bx1*, *Igl*, and *ZmTSAlike *have been described previously [17,18]. A search of the GeneBank and maize genomic database (see Availability and requirements section for URL) revealed putative TSA sequences, which do not constitute alleles of *Bx1*, *Igl*, or *ZmTSAlike*. These sequences, partly represented by the Tentative Unique Gene (TUG) *Z*mtuc03-08-11.4557, correspond to a new gene, now designated *ZmTSA. ZmTSA *is located on chromosome 7 (contig AC191027, GeneBank). A full-length *ZmTSA *cDNA clone was isolated [GenBank:EU334442]. Most plant TSAs have divergent N-terminal sequences that have no counterpart in bacteria and represent transit peptides for plastid import. When this variable part is excluded from the analysis, *Zm*TSA is 63% identical to BX1, 67% to IGL, and 72% to *Zm*TSAlike on protein level, respectively [for an alignment, see Additional file 1].

### Expression and subcellular localization

For a further characterization of the closely related genes *ZmTSA *and *ZmTSAlike *their transcription levels were determined by RT-PCR in different tissues and developmental stages. *ZmTSA *and *ZmTSAlike *transcripts were detected in all 16 tissues analyzed [see Additional file 2] and generally, *ZmTSA *was the predominant isoform expressed. The average transcript levels relative to *GAPDH *in these preparations were determined as 1.80 ± 0.93 fg fg^-1 ^for *Zm*TSA and 0.20 ± 0.15 fg fg^-1 ^for *Zm*TSA*like*. The virtually homogeneous expression of *ZmTSA *and *ZmTSAlike *qualifies them as candidates to function in tryptophan biosynthesis in maize. In contrast, *Bx1 *is predominantly transcribed in seedlings and *Igl *is specifically induced in response to herbivore attack [17,18,20].

There is a number of data suggesting that plant tryptophan biosynthesis is predominantly localized in the plastids [13,29]. *Zm*TSA and *Zm*TSAlike were analyzed *in silico *for putative targeting sequences using "TargetP" and "iPSORT" [22,23]. *Zm*TSA was predicted to be plastid localized, while *Zm*TSAlike, 45 amino acids shorter at the N-terminus, was expected to be retained in the cytoplasm.

**Figure 2:**
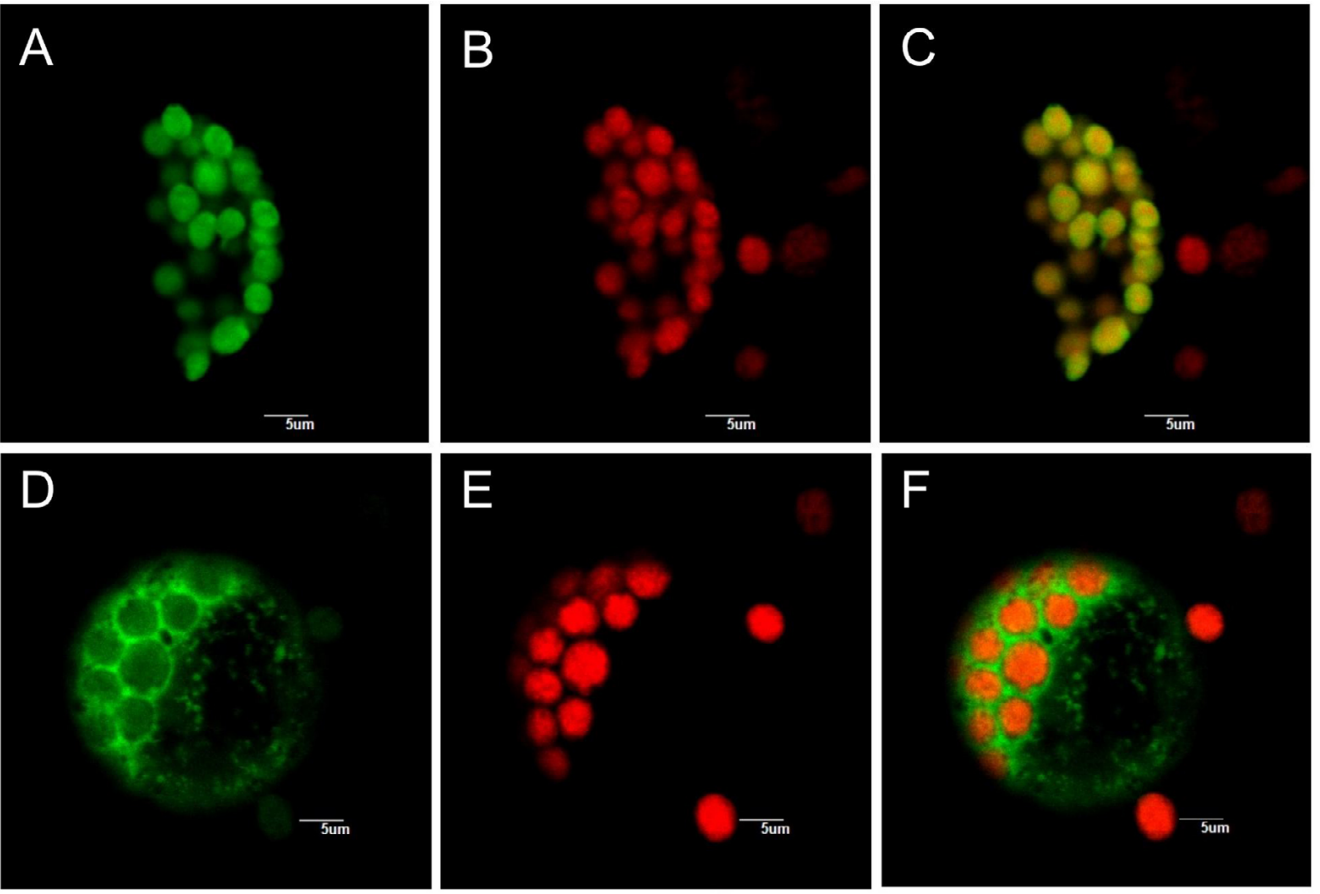
**Subcellular localization of *Zm*TSA and *Zm*TSAlike fused to eGFP in maize cells.** Analysis of maize mesophyll protoplasts transiently expressing *Zm*TSA::eGFP (A-C) or *Zm*TSAlike::eGFP (D-F). A, D: GFP fluorescence; B, E: Red chlorophyll autofluorescence in chloroplasts; C, F: Merged images.

To obtain experimental evidence, plasmids conferring expression of *Zm*TSA- and *Zm*TSAlike-GFP fusion proteins were transformed into maize protoplasts. GFP and chlorophyll autofluorescence were analyzed by confocal microscopy (Figure 2). In case of *Zm*TSA GFP fluorescence coincided with the chlorophyll autofluorescence of the chloroplasts (Figure 2A–C), demonstrating plastidic localization of *Zm*TSA. In contrast, *Zm*TSAlike-GFP was localized in the cytosol (Figure 2D–F).

### ZmTSA has tryptophan synthase α activity

We determined the kinetic parameters for the TS α-reaction for the two candidates. Purified recombinant *Zm*TSA was tested for conversion of IGP to indole. A low, but clearly detectable IGP turnover (K_cat _= 0.006 s^-1^) was observed (Table 1). No α-activity of recombinant *Zm*TSAlike was detected in analogous experiments.

**Table 1:** Kinetic parameters of *Zea mays *tryptophan synthase and comparison with characterized homologs.

Reaction	Recombinant Enzyme(s)	Substrate	K_M _(μM)	k_cat _(s^-1^)	k_cat_/K_M_(mM^-1 ^s^-1^)
α	*Zm*TSA	IGP	458 ± 94	0.006 ± 0.002	0.013
α	*Zm*TSA+*Zm*TSB1	IGP	52 ± 6	0.019 ± 0.004	0.37
β	*Zm*TSB1	Indole	24 ± 4	0.29 ± 0.04	12.1
β	*Zm*TSA+*Zm*TSB1	Indole	28 ± 6	0.44 ± 0.07	15.7
αβ	*Zm*TSA+*Zm*TSB1	IGP	47 ± 9	0.45 ± 0.06	9.6
α	*Zm*TSAlike	IGP	n. d.	n. d.	n. d.
α	*Zm*TSAlike+*Zm*TSB1	IGP	n. d.	n. d.	n. d.

α	BX1 ^[18]^	IGP	13	2.8	215
α	IGL ^[17]^	IGP	100	2.3	23

α	*Ec*TSA ^1)^	IGP	480	0.002	0.004
α	*Ec*TSA + *Ec*TSB ^1)^	IGP	27	0.2	7.4
α	*St*TSA + *St*TSB ^2)^	IGP	100	0.14	1.4
β	*St*TSA + *St*TSB ^2)^	Indole	15	3.6	240
αβ	*St*TSA + *St*TSB ^2)^	IGP	20	3	150

1) [31], *Ec*: *E. coli*.

2) [35], *St*: *S. typhimurium*.

n. d.: For *Zm*TSAlike no IGP turnover was detected.

### Formation of a tryptophan synthase complex *in vitro*

**Figure 3:**
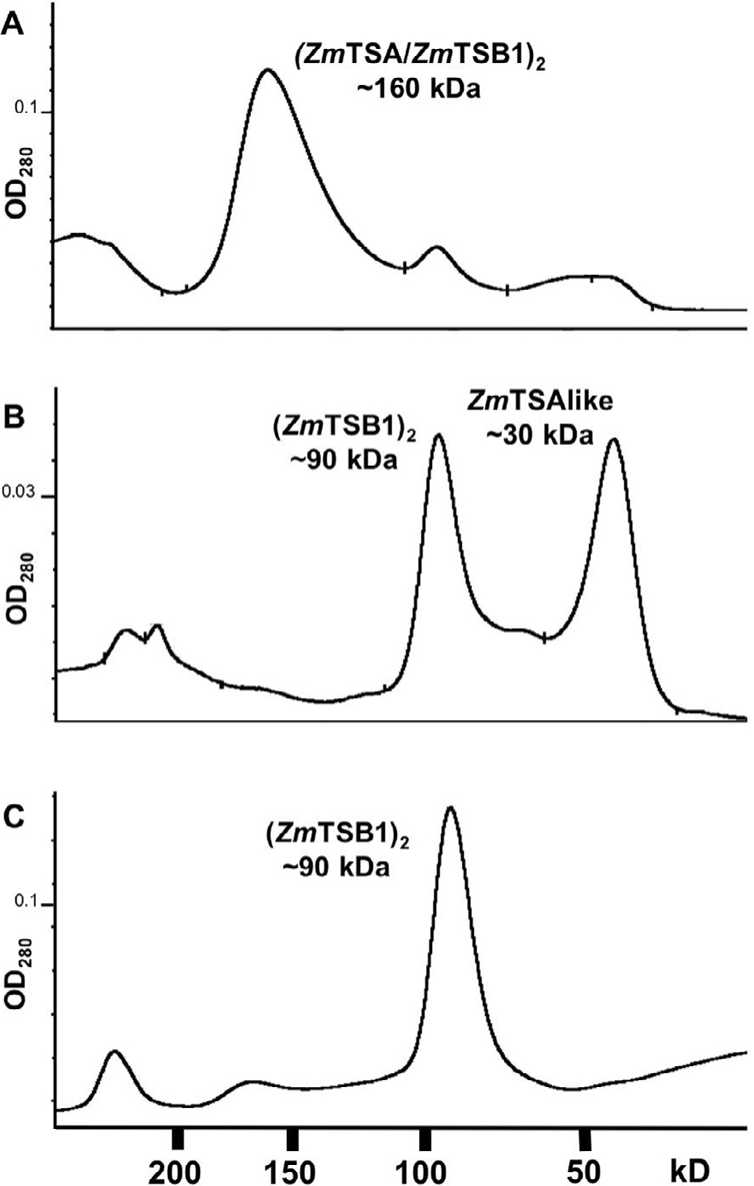
***In vitro *****complex formation**. Combinations of recombinant proteins, 0.5 mg each, were allowed to assemble 1 h at 4°C. An aliquot each was analyzed by size exclusion chromatography and the elution of protein was monitored by absorption at 280 nm. A: *Zm*TSA + *Zm*TSB1, the retention volume of the major peak corresponds the size of an α_2_β_2 _tetramer. B: *Zm*TSAlike + *Zm*TSB1, two peaks corresponding an α monomer and a β_2 _dimer are observed. C: *Zm*TSB1 without addition of a TSA homolog; putative β_2 _dimers are formed.

In bacteria α activity of the TS complex is two orders of magnitude higher than that of TSA alone. Therefore, *Zm*TSA was allowed to interact with purified recombinant *Zm*TSB1 for 1 h at 4°C adding 60 mM serine and 50 μM pyridoxal phosphate. To investigate complex formation with *Zm*TSB1*in vitro*, the *Zm*TSA/*Zm*TSB1 mixture was subjected to size exclusion chromatography (Figure 3). *Zm*TSA and *Zm*TSB1 formed a complex of approximately 160 kD (Figure 3A) that according to SDS-PAGE analysis (data not shown) contained both proteins in a 1:1 stoichiometry. These results are consistent with formation of a *Zm*TSA_2_*Zm*TSB1_2 _heterotetramer. The kinetic parameters of *Zm*TSA, *Zm*TSB1, and *Zm*TSA/*Zm*TSB1 heteromers were determined (Table 1). Heteromerisation with *Zm*TSB1 resulted in a 32-fold increase of the catalytic efficiency of *Zm*TSA.

No interaction of *Zm*TSB1 with *Zm*TSAlike, BX1, or IGL was detectable. The native molecular masses of *Zm*TSAlike, BX1, and IGL were estimated by gel filtration to around 30 kD, indicating that these proteins were monomers in solution (Figure 3B and data not shown). *Zm*TSB1 apparently formed dimers of approximately 90 kD (Figure 3B,C). No prominent larger complexes were observed. *Zm*TSAlike remained inactive with IGP as substrate also when the β-subunit was added to the preparation (Table 1). The experiment was repeated with thrombin digested proteins to exclude lack of complexation due to sterical hindrance caused by the His-tag yielding identical results (data not shown).

### Tryptophan synthase activity in leaf protein extracts

**Figure 4:**
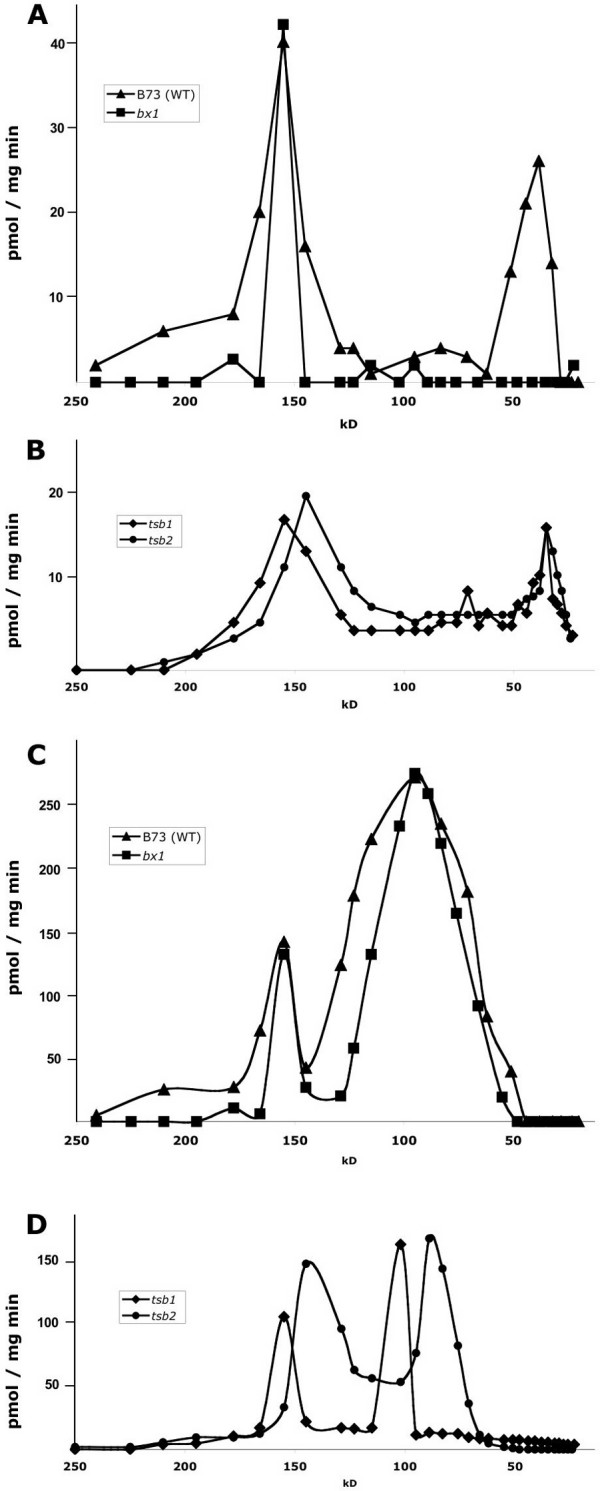
**Determination of enzymatic activities in leaf extracts fractionized by size exclusion chromatography.** Formation of indole from IGP (α-reaction, A, B) and of tryptophan from indole + serine (β-reaction, C, D) was quantified. The wildtype line B73, *bx1 *mutant (A, C), and *tsb1 *and *tsb2 *mutants (B, D) were analyzed.

To investigate, whether α_2_β_2 _TS complexes are also formed *in vivo*, protein extracts of B73 wildtype maize and the mutant lines *bx1*, *Zmtsb1*, and *Zmtsb2 *were separated by size exclusion chromatography. Individual fractions were tested for conversion of IGP to indole + glyceraldehyde-3-phosphate (α reaction) and of indole + serine to tryptophan (β reaction) (Figure 4). In a fraction representing proteins of approx. 160 kD both α- and β-activity was detected (app. K_M_^IGP ^= 47 ± 7 μM; app. K_M_^indole ^= 5 ± 2 μM, B73). This fraction was as well capable of the total (αβ) TS reaction in all genotypes tested (IGP to tryptophan turnover rates of 96 to 122 pmol mg^-1 ^min^-1^). β-Activity was also clearly detected in the fraction of approx. 90 kD proteins, the size of putative β-dimers. The *Zmtsb1 Zmtsb2 *(*orp1 orp2*) double mutant is devoid of β-activity and shows severe growth defects [15]. Here, the respective single mutants *Zmtsb1 *(*orp1 +/orp1 orp2*) and *Zmtsb2 *(*orp1 orp2/+ orp2*) were tested and each yielded β-activity in both the 90 kD and the 160 kD complex fractions (Figure 4D). This indicates that *Zm*TSB1 and *Zm*TSB2 are functionally redundant and may both form active β-dimers as well as active αββα TS complexes. In extracts from B73 wildtype, as well as *Zmtsb1 *or *Zmtsb2 *mutants a second α-activity peak was determined in a fraction of approximately 30 kD, corresponding to the monomer size of TSA homologs. In extracts of *bx1 *mutants this activity was not present, indicating that monomeric α-activity in leaves is predominantly due to activity of BX1 enzyme.

### *Zm*TSA is a component of the tryptophan synthase complex in maize

To identify constituents of the 160 kD TS complex *in planta *a mass-spectrometry-based approach was applied. Sequence qualifying peptides, i.e. peptides allowing annotation, were obtained for *Zm*TSA, *Zm*TSAlike, BX1, IGL, and *Zm*TSB1 by the analysis of tryptic digests of recombinant proteins using liquid chromatography with time-of-flight mass spectrometry coupled by electrospray ionisation (LC-ESI-ToF-MS). The resulting detection signals of peptides were compared with theoretically expected tryptic peptide masses [see Additional files 3 and 4]. 46.0% of the total sequence was covered for *Zm*TSA, 36.5% for *Zm*TSAlike, 40.4% for *Zm*TSB1, 50.0% for BX1, and 56.9% for IGL.

To identify the active α-subunit of the TS complex in maize, TS activity was partially purified from leaf extracts by subsequent ion exchange and size exclusion chromatography. The 160 kD protein fraction was concentrated and separated by SDS-PAGE. Proteins between approx. 25 kD (smaller than TSA size) and 60 kD (larger than TSB size) were cut out of the gel. A tryptic digest of these proteins was analyzed by LC-ESI-ToF-MS and surveyed for peptides sequence qualifying for *Zm*TSA, *Zm*TSAlike, BX1, IGL, and *Zm*TSB1.

**Figure 5:**
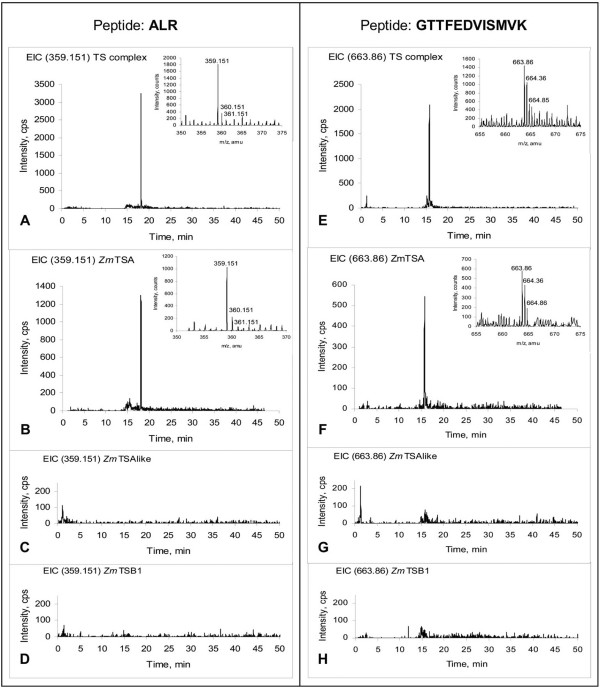
**Identification of *Zm*TSA as component of the tryptophan synthase complex.** The approach is exemplified by the peptides ALR (A-D, m/z = 359.151) and GTTFEDVISMVK (E-H, m/z = 663.860). These fragments are characteristic for *Zm*TSA (B, F) and were absent in *Zm*TSAlike (C, G) and *Zm*TSB1 (D, H). Both fragments were identified in a trypsin hydrolysate of the active tryptophan synthase fraction (A, E). The peptide and the corresponding ^13^C/^34^S isotopic peaks were detected (inserts in A, B, E, F).

Four peptides characteristic for *Zm*TSA were present as major peptide signals in this tryptic digest of the 160 kD fraction containing active TS (Table 2, Figure 5). The probability for a specific random dodeca-peptide, such as e.g. identified GTTFEDVISMVK is in the order of approximately 10^-15^. Therefore, *Zm*TSA was conclusively identified as component of a maize TS complex. No peptides specific for *Zm*TSAlike, BX1, or IGL were detected [see Additional file 3]. The peptides ADGTGPLIYLK and DATSEAIR were identified, which could be assigned to either of the highly similar active *Zm*TSB isoforms. For *Zm*TSB1 the specific peptide QALNVFR was identified. No *Zm*TSB2 specific peptide was clearly assigned. However, based on *Zmtsb1 *mutant analysis (Figure 4) it is very likely that also *Zm*TSB2 is present in TS complexes *in vivo*. In summary, *Zm*TSA and *Zm*TSB1 were identified by LC-ESI-ToF-MSas constituents of TS complexes.

**Table 2:** *Zm*TSA sequence qualifying peptides identified in a tryptic digest of tryptophan synthase partially purified from leaves.

*ret. time (min)*	*m/z*	*charge*	*EIC intensity*	*calc. mass*	*peptide*
15.1	371.239	1+	5.60E+03	370.449	AALP
15.5	687.361	2+	7.00E+04	1373.480	TLEEAASPEEGLK
15.7	663.860	2+	5.00E+02	1326.528	GTTFEDVISMVK
18.2	359.151	1+	1.30E+03	359.440	ALR

EIC: Extracted ion chromatogram

## Discussion

Evidence for physical interaction of tryptophan synthase α and β subunits in plants was provided by immunoaffinity chromatography for Arabidopsis [14] and by size exclusion chromatography for maize (this study). For the identification of the maize tryptophan synthase components *in vivo*, specific sequence qualifying peptides were assigned by LC-MS. This approach allows the analysis of enzyme complexes that are not sufficiently stable for application of a larger variety of chromatographic separations, necessary for purification to homogeneity. Applying this method, *Zm*TSA was identified as α-subunit of a tryptophan synthase complex.

The apparent molecular weight strongly indicates that in maize tryptophan synthase is functional as αββα heterotetramer, similar as in bacteria [30,31]. In maize *Zm*TSA is the principal α-subunit of the complex. Catalytic efficiency of *Zm*TSA was enhanced more than 30-fold by interaction with the β-subunit (Table 1). Such activating interaction between α- and β-subunit is also well known from bacteria like *E. coli *(Table 1). In *E. coli *this activation was mutual, i.e. β-activity strongly increased upon formation of an α_2_β_2 _TS complex [32]. In contrast, no significant activation of the maize β-subunit by *Zm*TSA was observed (Table 1). The reason for this difference between the bacterial and plant enzyme is not known.

In maize, ancestral TSAs have been recruited for secondary metabolism. The TSA homologs BX1 and IGL catalyze the formation of indole, which functions as DIMBOA precursor or volatile signal [17-19] (Table 1) and BX1 monomer activity was observed in leaf extracts (Figure 4). In addition, TSB dimers that have been observed in leaf extracts (Figure 4) and *in vitro *(Figure 3), which possibly function in salvage of indole by its conversion to tryptophan. It remains open, whether a mechanism involving BX1 monomer and TSB dimer contributes significantly to the total metabolic flux towards tryptophan. The *bx1 *mutant and the *bx1 igl *double mutant (M. Frey, unpublished data) are fully viable; therefore this process is not essential at any stage of development.

We propose that, despite the availability of the highly active monomers BX1 and IGL, in maize tryptophan is predominantly synthesized through a tryptophan synthase complex containing *Zm*TSA and *Zm*TSB1 or *Zm*TSB2, respectively. Probably this complex has been retained during evolution for tryptophan synthesis, as it enables substrate channelling and allosteric regulation. Knockout mutants could serve as ultimate proof for *Zm*TSA being essential. Therefore we have extensively screened public databases as well as the large Pioneer HiBred TUSC collection for *Mu*-transposon insertion mutants [33] in *Zm*TSA. However, no insertion alleles were identified. It remains open, whether *Zm*tsa knockout mutants are lethal.

Based on import studies, subcellular fractionation, and target sequence prediction, it is suggested that in plants the biosynthesis of aromatic amino acids is predominantly localized in plastids (for review, see [29]). Consistent with these data *Zm*TSA contains a chloroplast targeting sequence and *Zm*TSA::GFP fusion proteins were targeted to the plastid (Figure 2). Interestingly, the TSA homolog *Zm*TSAlike lacks such a transit peptide and *Zm*TSAlike::GFP fusion proteins were located in the cytosol (Figure 2). It has been debated whether the biosynthesis of aromatic amino acids is also partially active in the cytoplasm, as e.g. a cytoplasmic isoform of chorismate mutase is expressed in Arabidopsis [34]. As recombinant *Zm*TSAlike, expressed in *E. coli*, did not show any α-activity it remains unclear, whether *Zm*TSAlike functions as cytosolic TSA isoform. *Zm*TSAlike might either require specific conditions and modifications or it has an unknown function in plant metabolism.

## Conclusion

Four TSA homologs exist in maize. Only one of these isoforms, *Zm*TSA, is involved in the formation of a tryptophan synthase complex. Based on our data and previous results for *Arabidopsis thaliana *we propose that a ubiquitous tryptophan synthase complex is responsible for tryptophan formation in angiosperms, like in fungi and bacteria.

## Abbreviations

ACN: acetonitrile; *bx1: benzoxazinless1*; GAPDH: glyceraldehyde-3-phosphate dehydrogenase; IGL: indole-3-glycerol phosphate lyase; IGP: indole-3-glycerol phosphate; TSA: tryptophan synthase alpha subunit; TSB: tryptophan synthase beta subunit; wap: weeks after pollination.

## Availability and requirements

Maize genomic database: 

## Authors' contributions

VK designed, conducted, and analyzed the majority of experiments and supported drafting the manuscript. LW carried out the LC-MS analysis. AF analyzed the sub-cellular localisation of proteins. TL designed and supervised the LC-MS analysis and interpretation. MF conducted transcription analysis, supervised localisation studies and cloning, and revised the manuscript. AG conceptualised the project, coordinated the group, and revised the manuscript. EG supervised the project, supported experiment design and analysis, and wrote the draft of the manuscript. All authors read and approved the manuscript.

## Supplementary Material

Additional file 1 **Figure SF1**: Amino acid sequence alignment of the maize TSA homologs (ClustalW). Transit peptides are depicted in italics. For *Zm*TSA and IGL they were predicted using TargetP [23], for BX1 it was determined experimentally [36]. Click here for file

Additional file 2 **Table S1**: Transcript levels of *ZmTSA *and *ZmTSAlike *relative to *GAPDH *in RNA preparations from different tissues. Click here for file

Additional file 3 **Table S2**: MS-signals yielding identification of sequence qualifying peptides of recombinant *Zm*TSA, *Zm*TSAlike, IGL, BX1, and *Zm*TSB1. Click here for file

Additional file 4 **Table S3**: LC/MSD TOF analysis of trypsin digested recombinant *Zm*TSA, *Zm*TSAlike, IGL, BX1, and TSB1; list of major signals. Click here for file
