# Supplementary material for: Characterisation of the tryptophan synthase alpha subunit in maize
Source: BMC Plant Biol. 2008 Apr 22;8:44. doi: 10.1186/1471-2229-8-44 (PMC2395261; doi:10.1186/1471-2229-8-44)
Supplement: Additional file 1 — Figure SF1: Amino acid sequence alignment of the maize TSA homologs (ClustalW). Transit peptides are depicted in italics. For ZmTSA and IGL they were predicted using TargetP [23], for BX1 it was determined experimentally [36]. [file 1471-2229-8-44-S1.doc]

| Tissue | ZmTSA/GAPDH | *ZmTSAlike*/*GAPDH* |
| --- | --- | --- |
| leaf 12 d | 2.14 | 0.10 |
| leaf 12 d MeJA | 2.26 | 0.13 |
| leaf 12 d elicitor | 2.13 | 0.17 |
| shoot 4d | 0.88 | 0.10 |
| shoot 6d | 0.95 | 0.08 |
| shoot light 6d | 0.87 | 0.05 |
| root 3 weeks | 1.90 | 0.17 |
| crownroot 10 weeks | 0.78 | 0.13 |
| stem 8 weeks | 1.17 | 0.32 |
| leaf 10 weeks | 2.05 | 0.07 |
| husk 10 weeks | 1.14 | 0.25 |
| silk 10 weeks | 1.83 | 0.46 |
| cob | 2.23 | 0.26 |
| kernel 1 wap | 2.29 | 0.61 |
| kernel 3 wap | 4.55 | 0.24 |
| tassel | 1.54 | 0.13 |
