# Supplementary material for: Characterisation of the tryptophan synthase alpha subunit in maize
Source: BMC Plant Biol. 2008 Apr 22;8:44. doi: 10.1186/1471-2229-8-44 (PMC2395261; doi:10.1186/1471-2229-8-44)
Supplement: Additional file 2 — Table S1: Transcript levels of ZmTSA and ZmTSAlike relative to GAPDH in RNA preparations from different tissues. [file 1471-2229-8-44-S2.doc]

**MS-signals yielding identification of sequence qualifying peptides of recombinant *Zm*TSA, *Zm*TSAlike, IGL, BX1, and *Zm*TSB1.** Sequence qualifying peptides that were identified in partially purified tryptophan synthase from maize leaves are printed in bold. As example, the signals allowing identification of the peptides ALR and GTTFEDVISMVK are shown in Fig. 5.

***Zm*TSA**

| *ret. time* | *m/z* | *charge* | *EIC intensity* | *calc. mass* | *peptide* |
| --- | --- | --- | --- | --- | --- |
| 14.1 | 1123.120 | 2+ | 1.20E+04 | 2244.530 | EAGVHGLVVPDVPLEETDVL |
| 14.5 | 670.700 | 3+ | 3.20E+04 | 2009.293 | VTEKPVAVGFGVSTPEHVR |
| 14.5 | 1005.536 | 2+ | 3.20E+03 | 2009.081 | VTEKPVAVGFGVSTPEHVR |
| 14.6 | 540.797 | 2+ | 5.60E+04 | 1080.206 | SISGTFAELR |
| 14.6 | 1080.562 | 1+ | 4.40E+04 | 1080.206 | SISGTFAELR |
| 14.7 | 886.468 | 2+ | 5.00E+03 | 1773.080 | QIAGWGADGVIIGSAVMK |
| 14.9 | 642.689 | 3+ | 6.40E+03 | 1925.169 | NNLELVLLTTPTTPNER |
| **15.1** | **371.239** | **1+** | **5.60E+03** | **370.449** | **AALP** |
| 15.2 | 963.536 | 2+ | 8.00E+03 | 1925.169 | NNLELVLLTTPTTPNER |
| 15.4 | 749.068 | 3+ | 3.80E+04 | 2244.530 | EAGVHGLVVPDVPLEETDVL |
| **15.5** | **687.361** | **2+** | **7.00E+04** | **1373.480** | **TLEEAASPEEGLK** |
| **15.7** | **663.860** | **2+** | **5.00E+02** | **1326.528** | **GTTFEDVISMVK** |
| **18.2** | **359.151** | **1+** | **1.30E+03** | **359.440** | **ALR** |

***Zm*TSAlike**

| *ret. time* | *m/z* | *charge* | *EIC intensity* | *calc. mass* | *peptide* |
| --- | --- | --- | --- | --- | --- |
| 15.1 | 701.368 | 1+ | 1.80E+04 | 701.781 | SNVNLR |
| 15.2 | 417.243 | 3+ | 1.70E+04 | 1250.417 | LTVAETFSNLR |
| 15.4 | 424.223 | 2+ | 1.00E+03 | 846.957 | MANGGAAAGK |
| 15.5 | 656.706 | 3+ | 4.00E+04 | 1967.253 | VTDKPVAVGFGVSTPEHVK |
| 15.5 | 984.525 | 2+ | 7.00E+03 | 1967.253 | VTDKPVAVGFGVSTPEHVK |
| 15.9 | 527.264 | 2+ | 1.90E+04 | 1053.241 | GVGNFMSTIK |
| 16.0 | 626.351 | 2+ | 1.50E+04 | 1250.417 | LTVAETFSNLR |
| 16.1 | 885.510 | 2+ | 1.00E+03 | 1769.074 | QIVGWGADGVIVGSAIVK |
| 16.3 | 654.359 | 2+ | 4.50E+04 | 1306.537 | GTTLDSVIEMLK |
| 16.9 | 352.214 | 3+ | 2.10E+04 | 1053.241 | GVGNFMSTIK |
| 17.0 | 590.406 | 3+ | 1.20E+04 | 1769.074 | QIVGWGADGVIVGSAIVK |
| 17.3 | 516.350 | 3+ | 1.30E+04 | 1545.684 | QLCEAATPEEGLER |

**IGL**

| *ret. time* | *m/z* | *charge* | *EIC intensity* | *calc. mass* | *peptide* |
| --- | --- | --- | --- | --- | --- |
| 15.0 | 778.407 | 1+ | 1.29E+03 | 777.891 | MEEITR |
| 15.1 | 197.119 | 2+ | 6.58E+03 | 392.474 | MSR |
| 15.1 | 389.213 | 2+ | 3.07E+03 | 777.891 | MEEITR |
| 15.3 | 144.927 | 2+ | 1.38E+04 | 287.362 | LR |
| 15.3 | 393.208 | 1+ | 4.47E+02 | 392.474 | MSR |
| 15.3 | 773.405 | 2+ | 4.53E+03 | 1544.827 | ALASGTTPDGVLAMLK |
| 15.3 | 625.304 | 3+ | 4.04E+04 | 1873.157 | QIAEWGADGVIIGSAMVR |
| 15.4 | 288.181 | 1+ | 1.28E+03 | 287.362 | LR |
| 15.4 | 561.308 | 1+ | 8.27E+03 | 560.648 | TEAIK |
| 15.4 | 522.292 | 2+ | 1.58E+03 | 1043.228 | VQSLIQEVK |
| 15.5 | 799.409 | 3+ | 3.95E+03 | 2395.759 | EAGVHGLIVPDLPYGNSCALTLR |
| 15.6 | 337.203 | 2+ | 5.76E+03 | 673.727 | ANVNTR |
| 15.6 | 575.318 | 2+ | 4.86E+02 | 1148.327 | WGLADFAAAVK |
| 15.7 | 674.366 | 1+ | 1.55E+03 | 673.727 | ANVNTR |
| 15.8 | 333.184 | 1+ | 2.94E+03 | 332.360 | ASR |
| 15.9 | 645.353 | 1+ | 6.70E+04 | 644.743 | NALPCQ |
| 16.1 | 610.324 | 3+ | 3.98E+03 | 1827.065 | NSLELVLLTTPSTPADR |
| 16.2 | 166.050 | 2+ | 1.12E+06 | 332.360 | ASR |
| 16.2 | 451.237 | 2+ | 6.99E+03 | 899.999 | QLGEAASPK |
| 16.2 | 776.407 | 2+ | 2.42E+03 | 1550.778 | GFVYLATVNGVTGPR |
| 16.4 | 914.488 | 2+ | 5.28E+03 | 1827.065 | NSLELVLLTTPSTPADR |

**BX1**

| *ret. time* | *m/z* | *charge* | *EIC intensity* | *calc. mass* | *peptide* |
| --- | --- | --- | --- | --- | --- |
| 15.1 | 1045.592 | 1+ | 1.20E+03 | 1044.213 | VESLIQEVK |
| 15.1 | 523.299 | 2+ | 4.50E+03 | 1044.213 | VESLIQEVK |
| 15.1 | 840.410 | 2+ | 4.50E+02 | 1678.980 | ALASGTTMDAVLEMLR |
| 15.4 | 670.343 | 1+ | 2.50E+03 | 669.738 | ANVNPR |
| 16.0 | 927.492 | 2+ | 4.50E+03 | 1852.077 | ASEGFVYLVSVNGVTGPR |
| 16.0 | 618.319 | 3+ | 2.40E+03 | 1852.077 | ASEGFVYLVSVNGVTGPR |
| 16.0 | 675.365 | 3+ | 1.40E+03 | 2022.286 | NNNLELVLLTTPAIPEDR |
| 16.0 | 1012.038 | 2+ | 1.20E+03 | 2022.286 | NNNLELVLLTTPAIPEDR |
| 16.0 | 1012.038 | 3+ | 1.20E+03 | 2660.025 | EAGVHGLIVPDLPYVAAHSLWSEAK |
| 16.1 | 1086.555 | 3+ | 5.00E+02 | 3257.721 | LLDGCGADVIELGVPCSDPYIDGPIIQASVAR |
| 16.3 | 489.322 | 1+ | 3.90E+02 | 489.569 | EITK |
| 17.5 | 560.394 | 3+ | 3.60E+02 | 1678.980 | ALASGTTMDAVLEMLR |
| 17.6 | 335.179 | 1+ | 3.00E+03 | 334.434 | GMK |

**TSB**

| *ret. time* | *m/z* | *charge* | *EIC intensity* | *calc. mass* | *peptide* | *isoform* |
| --- | --- | --- | --- | --- | --- | --- |
| 14.5 | 509.266 | 1+ | 3.00E+05 | 508.572 | YLDV | 1+2 |
| 14.5 | 492.777 | 2+ | 1.18E+04 | 984.200 | AVHSGTATLK | 1+2 |
| 14.6 | 492.775 | 2+ | 2.90E+04 | 984.120 | AVHSGTATLK | 1+2 |
| 14.6 | 901.918 | 2+ | 2.80E+04 | 1801.881 | AEYDSVTDQEALDAFK | 1+2 |
| 14.8 | 741.402 | 2+ | 6.00E+04 | 1481.626 | LIGVEAAGHGVDTDK | 1 |
| 14.8 | 741.902 | 1+ | 4.00E+04 | 740.858 | HAATLTK | 1+2 |
| 14.8 | 690.400 | 3+ | 2.70E+04 | 2068.132 | LEGIIPALETSHALAYLEK | 1+2 |
| 14.8 | 535.800 | 2+ | 4.50E+03 | 1070.272 | LCPTLPDGVR | 1 |
| 14.9 | 787.460 | 1+ | 4.00E+04 | 786.924 | ELDGILK | 1+2 |
| 14.9 | 601.288 | 3+ | 2.40E+03 | 1801.881 | AEYDSVTDQEALDAFK | 1+2 |
| 15.0 | 757.462 | 1+ | 9.80E+04 | 756.900 | LLGAEVR | 1+2 |
| 15.0 | 901.921 | 2+ | 2.40E+04 | 1801.881 | AEYDSVTDQEALDAFK | 1+2 |
| **15.2** | **423.233** | **2+** | **8.00E+02** | **846.985** | **QALNVFR** | **1** |
| 15.2 | 1035.173 | 2+ | 2.20E+03 | 2068.132 | LEGIIPALETSHALAYLEK | 1+2 |
| 15.4 | 556.281 | 2+ | 6.50E+03 | 1111.542 | ESPLYFAER | 1+2 |
| **15.4** | **574.315** | **2+** | **4.80E+03** | **1147.337** | **ADGTGPLIYLK** | **1+2** |
| **15.4** | **574.807** | **2+** | **1.23E+04** | **1147.337** | **ADGTGPLIYLK** | **1+2** |
| 15.5 | 613.853 | 2+ | 1.40E+04 | 1225.454 | INNAVAQALLAK | 1+2 |
| 15.5 | 741,374 | 2+ | 3.80E+04 | 1481.759 | LIGVEAAGHGVDTDK | 1 |
| 15.5 | 643.243 | 3+ | 1.00E+04 | 1925.178 | IIAETGAGQHGVATATVCAR | 1 |
| 15.6 | 613.856 | 2+ | 7.00E+03 | 1225,454 | INNAVAQALLAK | 1+2 |
| 15.6 | 690.398 | 3+ | 3.00E+03 | 2068.132 | LEGIIPALETSHALAYLEK | 1+2 |
| 15.7 | 1035.096 | 2+ | 3.00E+04 | 2068.398 | LEGIIPALETSHALAYLEK | 1+2 |
| **15.7** | **435.770** | **2+** | **1.00E+04** | **861.907** | **DATSEAIR** | **1+2** |
| 15.9 | 614.365 | 1+ | 7.00E+03 | 613.733 | QAMHK | 1 |

For *Zm*TSB1 peptides sequences present for both *Zm*TSB1 and *Zm*TSB2 isoforms “1+2” is denoted.
